# Effects of Algorithmic Music on the Cardiovascular Neural Control

**DOI:** 10.3390/jpm11111084

**Published:** 2021-10-25

**Authors:** Alfredo Raglio, Beatrice De Maria, Francesca Perego, Gianluigi Galizia, Matteo Gallotta, Chiara Imbriani, Alberto Porta, Laura Adelaide Dalla Vecchia

**Affiliations:** 1 IRCCS Istituti Clinici Scientifici Maugeri, 27100 Pavia, Italy; alfredo.raglio@icsmaugeri.it (A.R.); chiara.imbriani@icsmaugeri.it (C.I.); 2 IRCCS Istituti Clinici Scientifici Maugeri, 20138 Milan, Italy; beatrice.demaria@icsmaugeri.it (B.D.M.); francesca.perego@icsmaugeri.it (F.P.); matteo.gallotta@icsmaugeri.it (M.G.); 3 IRCCS Istituti Clinici Scientifici Maugeri, 28010 Veruno, Italy; gianluigi.galizia@icsmaugeri.it; 4 Department of Biomedical Sciences for Health, University of Milan, 20133 Milan, Italy; alberto.porta@unimi.it; 5 Department of Cardiothoracic, Vascular Anesthesia and Intensive Care, IRCCS Policlinico San Donato, San Donato Milanese, 20097 Milan, Italy

**Keywords:** cardiovascular neural control, heart rate variability, arterial pressure variability, baroreflex, music listening, algorithmic music, Melomics-Health

## Abstract

Music influences many physiological parameters, including some cardiovascular (CV) control indices. The complexity and heterogeneity of musical stimuli, the integrated response within the brain and the limited availability of quantitative methods for non-invasive assessment of the autonomic function are the main reasons for the scarcity of studies about the impact of music on CV control. This study aims to investigate the effects of listening to algorithmic music on the CV regulation of healthy subjects by means of the spectral analysis of heart period, approximated as the time distance between two consecutive R-wave peaks (RR), and systolic arterial pressure (SAP) variability. We studied 10 healthy volunteers (age 39 ± 6 years, 5 females) both while supine (REST) and during passive orthostatism (TILT). Activating and relaxing algorithmic music tracks were used to produce possible contrasting effects. At baseline, the group featured normal indices of CV sympathovagal modulation both at REST and during TILT. Compared to baseline, at REST, listening to both musical stimuli did not affect time and frequency domain markers of both SAP and RR, except for a significant increase in mean RR. A physiological TILT response was maintained while listening to both musical tracks in terms of time and frequency domain markers, compared to baseline, an increase in mean RR was again observed. In healthy subjects featuring a normal CV neural profile at baseline, algorithmic music reduced the heart rate, a potentially favorable effect. The innovative music approach of this study encourages further research, as in the presence of several diseases, such as ischemic heart disease, hypertension, and heart failure, a standardized musical stimulation could play a therapeutic role.

## 1. Introduction

The therapeutic use of music in its different forms is the subject of several studies in clinical and non-clinical fields [1,2,3,4,5,6,7,8,9]. However, the therapeutic applications of music require further scientific investigation aimed at validating the results and at standardizing the stimuli. Standardization is necessary to limit the heterogeneous individual responses to music listening and to relate specific musical parameters/structures to potential specific effects. In particular, standardization circumvents the cultural dimension of the musical listening experience avoiding references to precise musical styles, but also to biographical or personal aspects of the listener. In this direction, an algorithmic musical approach could be helpful in standardizing musical stimuli with the aim of obtaining reproducible and effective clinical outcomes. Music is associated with activity changes in brain structures known to involve several pathways, including the transmission of information into the cardiac nerve plexus, involving the autonomic and endocrine pathways, blood pressure and blood gases regulatory systems [10]. The influence of music on the autonomic cardiovascular (CV) parameters has been widely documented [2,3,4,8,11]. It is not clear whether different types of music or sound may produce different effects, or rather which specific effect corresponds to a particular musical stimulus. Thus, it is difficult to make musical choices to achieve specific outcomes. It is even harder to hypothesize clear therapeutic applications. Thus, a straightforward answer to the above queries is not easily available. First, the complexity and heterogeneity of musical structures [12], second, the broad interaction of music itself within the brain, the limbic, the paralimbic system, and the autonomic nervous system (ANS) [13], lastly, the limited availability of noninvasive approaches for the evaluation of the autonomic function [14] are the main issues to overcome. Few studies [3] have documented some controversial effects of musical listening on ANS when evaluated noninvasively via the heart rate variability analysis. These inconsistent findings could derive from the type of used musical stimuli, in particular the presence of emotional contents [3,15], the recipient’s personal musical taste, and the great methodological heterogeneity [3]. In the field of the therapeutic use of music, algorithmic music is composed using an algorithm based on predefined structures/parameters that the music therapist chooses to pursue therapeutic purposes. This has already been tested in patients with dementia, mood imbalance and in subjects with work-related stress [5,8,16].

In the present protocol, music was created with the specific goal of producing contrasting effects (activation/excitement versus relaxation/quiescence), based on specific sound parameters [12]. Autonomic response was assessed via time and frequency domain markers derived from beat-to-beat series of heart period, approximated as the time distance between two consecutive R-wave peaks (RR), and systolic arterial pressure (SAP). The autonomic state was evaluated during supine condition (REST) and head-up tilting test (TILT), i.e., a passive postural maneuver that causes cardiac sympathetic activation and vagal withdrawal to cope with postural hydrostatic changes. The impact of algorithmic music in these conditions has never been tested. Thus, our study aims to assess the effects of listening to algorithmic music on the CV neural regulation of healthy subjects by means of the spectral analysis of RR and SAP variability both at REST and during TILT.

## 2. Methods

### 2.1. Study Population

Ten healthy volunteers (age 39 ± 6 years, 5 females) were enrolled at IRCCS Istituti Clinici Scientifici Maugeri in Milan. The sample size was derived from previous studies assessing CV indices in young healthy subjects during rest and orthostasis [16,17]. At the screening assessment, a detailed interview and complete physical examination were performed. Inclusion criteria were: (i) healthy status; (ii) age between 30 and 50 years. Exclusion criteria were: (i) any cardiovascular, respiratory, metabolic or acute disease; (ii) any current pharmacological therapy known to influence the CV neural control; (iii) alcohol consumption >24 g/day (>250 mL of wine, >660 mL of beer, >80 mL of spirits); (iv) moderate to heavy smoking (>8 cigarettes daily).

### 2.2. Algorithmic Music Approach

The sound-musical stimuli used for this study were composed utilizing the algorithm Melomics-Health [12,18,19]. This way of composing music gives the possibility to define the design of music, setting the conditions for its therapeutic use. The music can be composed based on specific sound parameters and appropriate musical structures, in order to achieve the therapeutic goals. One of the advantages of this technology is precisely given by the possibility of adapting/shaping the music according to the therapeutic objective, leaving the algorithm to produce it. This approach has been recently supported as a novel therapeutic tool [12,19]. Accordingly, in this study, the algorithm Melomics-Health produced “songs” with either activating or relaxing content to verify whether the sympathetic and the parasympathetic branches of the ANS would be elicited in a manner similar to the motor system [20]. For this purpose, the *tempo*, musical values and melodic characteristics of the songs were predefined. For the activating algorithmic music track (Track1, example in Supplementary Materials), high frequency, high density, short values and short rhythmic/melodic variations were used to capture the subject’s attention, activating him/her on a psycho-physical level. On the contrary, for the relaxing algorithmic music tracks (Track2, example in Supplementary Materials), low frequency, low density, long values and long rhythmic/melodic variations were used to deactivate the subject [20]. Each piece of music varies the pitches of the sounds (they are all melodic lines, in fact), but each maintains its structure unchanged (activating or relaxing, depending on the case). Each sequence of songs was made of two pieces, one for cello and one for clarinet, each lasting 3 min. The final tracks had no references to specific styles or tonal rules; therefore, they could not be associated with existing musical pieces. This allowed the potential effect to be traced back mainly to the musical structure and parameters rather than to their cultural impact. All enrolled subjects were asked to listen to the tracks with their eyes closed as much as possible, wearing Bluetooth headphones.

### 2.3. Experimental Protocol

**Figure 1:**
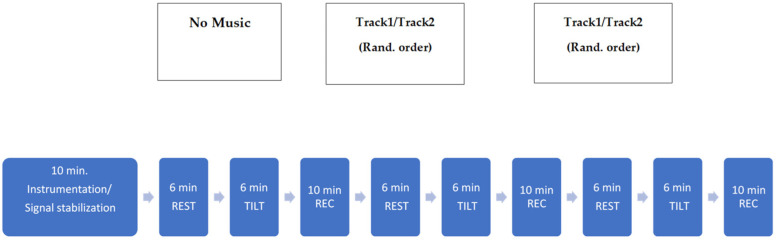
Track1, activating algorithmic music; Track2, relaxing algorithmic music; Rand., randomized; REST, supine condition; TILT, passive head-up tilting test; min., minutes; REC, Recovery Time.

All the subjects were studied in the morning, after a good night sleep, in a quiet room. They were asked to avoid alcoholic or caffeinated beverages in the 12 h preceding the test. Subjects were equipped to acquire the electrocardiogram (ECG, modified lead II), respiration (via a thoracic belt—Marazza, Monza, Italy) and non-invasive beat-to-beat AP, via a photopletismographic device (Finometer Midi, Finapress Medical System). The arm was fixed at the level of the heart to optimize the AP measurements. Signals were sampled at 500 Hz. The timeline of the protocol is shown in Figure 1.

After 10 min in supine rest to allow stabilization, the signals were continuously acquired during basal conditions (B), i.e., without any stimulus, but the orthostatic challenge, listening to Track1 and listening to Track2. The two sessions were randomized. Each session lasted 12 min: 6 min with the subject lying supine (REST) and 6 min during head-up tilt test at 70° (TILT). A recovery period of 10 min was performed in the supine position without any interference between one session and another, in order to restore baseline signal conditions.

This study was conducted in accordance with the rules of the Declaration of Helsinki of 1995, revised in 2013, and was approved by the local Ethics Committee (IRCCS Istituti Clinici Scientifici Maugeri Ethics Committee; approval number 2335CE, date of approval 10.09.2019). Each enrolled subject signed a written informed consent.

### 2.4. Cardiovascular Neural Control Assessment

The CV neural control was assessed using the analysis of the RR interval variability, the AP variability and the cardiac baroreflex sensitivity (cBRS).

The RR interval was approximated as the temporal distance between two consecutive R-wave peaks detected on the ECG signal. An automated algorithm detected the QRS complexes imposing a threshold on the first derivative of the ECG signal and fixed the R peaks by means of parabolic interpolation. From the AP signal, systolic AP (SAP) and diastolic AP (DAP) beat-to-beat time series were derived. SAP values were derived as the maximum of the AP signal inside each considered RR interval. DAP values were derived as the minimum of the AP signal inside each considered RR interval. The detection of R peaks, DAP and SAP values were visually checked and manually corrected in case of misdetection. In presence of artifacts or ectopic beats, parabolic interpolation was performed, paying attention to never exceed 5% of the total time series.

For each experimental session (B, Track1, Track2), selections of 300 consecutive and synchronized values of RR, SAP and DAP time series were selected at REST and TILT for further analysis, in accordance with the rules for short-term variability analysis [21]. After linear detrending of the series, the mean of RR, SAP and DAP series (μ_RR_, μ_SAP_, μ_DAP_, respectively) were calculated and expressed in ms and mmHg. The variance of RR and SAP series were calculated as well (σ^2^_RR_ and σ^2^_SAP_, respectively) and expressed in ms^2^ and mmHg^2^. Parametric power spectral analysis was performed on the RR and SAP time series to assess the CV neural control. The RR and SAP series were modelized by an autoregressive model, whose order was chosen according to Akaike criteria and decomposed into power spectral components. The power spectral components were classified as low frequency (LF) or high frequency (HF), if their central frequency dropped in the LF (0.04–0.15 Hz) or HF (0.15–0.4 Hz) band, respectively [21]. The sum of the absolute power of LF or HF power spectral components of RR series were labeled as LF_a,RR_ and HF_a,RR_, respectively, and expressed in ms^2^. LF_a,RR_ and HF_a,RR_ were also expressed in normalized units and labeled as LF_nu,RR_ and HF_nu,RR_, respectively. LF_nu,RR_ and HF_nu,RR_ were obtained by dividing LF_a,RR_ and HF_a,RR_, respectively, by the total variance diminished by the power of the very low frequency band (below 0.4 Hz) and then multiplying the results by 100. HF_a,RR_ and HF_nu,RR_ were considered as markers of the vagal modulation directed to the sinus node [14], while LF_a,RR_ and LF_nu,RR_ as indices providing some information about the sympathetic modulation directed to the sinus node [17,22]. The ratio between LF_a,RR_ and HF_a,RR_ was also calculated and labeled as LF/HF. As to the SAP series, the sum of the absolute power of the LF power spectral components was labeled as LF_SAP_, expressed in mmHg^2^, and taken as an index of the sympathetic modulation directed to the vessels [22].

We estimated the cBRS by means of the sequence method [23,24]. The method is based on the search of sequences of baroreflex origin over the RR and SAP series. A sequence was defined when the simultaneous increase or decrease of both RR and SAP for four consecutive heart beats occurred. The slope of the regression line over each found sequence was calculated and subsequently averaged over all sequences. This average was taken as an index of the cBRS and expressed in ms/mmHg.

All RR and SAP variability parameters are summarized in Table 1.

### 2.5. Statistical Analysis

Two-way repeated measures analysis via Wilcoxon signed rank test was applied to test the difference between the CV control indices in the two experimental conditions (i.e., REST and TILT) during the three different experimental sessions (B, Track1, Track2). The level of significance of each pairwise comparison was lowered according to the total number of tests (i.e., 6) to deal with the multiple comparison issue. A *p* < 0.05 was considered as significant. Data are expressed as mean ± standard deviation. The statistical analysis was carried out using the statistical program Sigmaplot (Systat Software, Chiacago, IL, USA, version 11.0).

## 3. Results

All the enrolled subjects completed the protocol without symptoms. Table 2 summarizes the main characteristics of the population.

**Figure 2:**
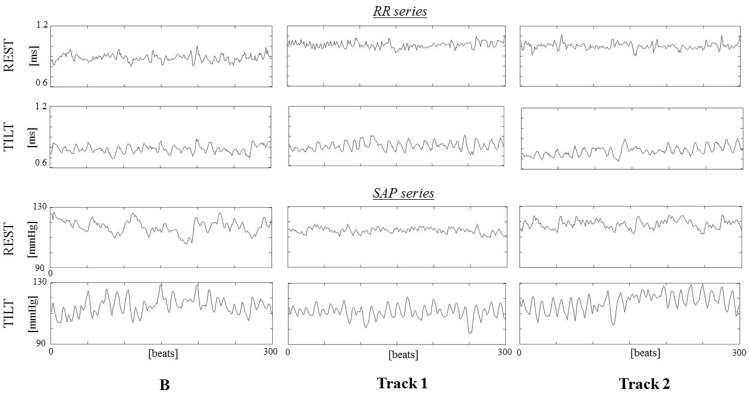
Example of RR (**upper panels**) and SAP (**lower panels**) series for each studied condition (B, baseline on the left, Track1 in the middle and Track2 on the right) during resting (REST, line 1 and 3 from the top) and tilting (TILT, line 2 and 4 from the top).

A representative example of RR and SAP series for each experimental phase is shown in Figure 2**.**

**Figure 3:**
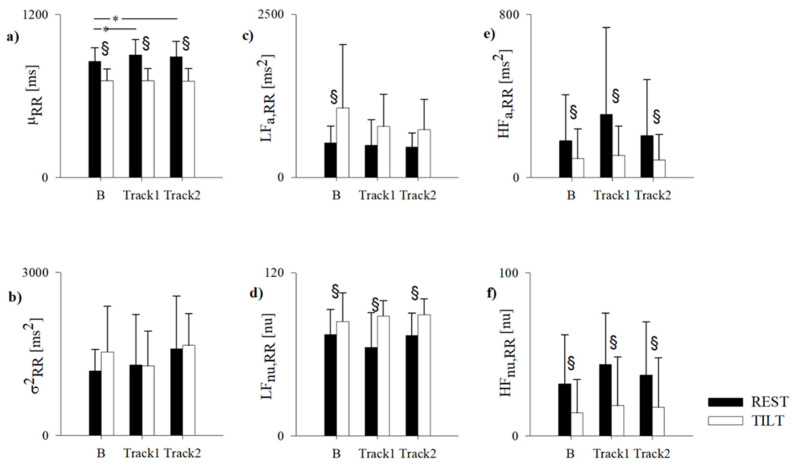
Results of the RR variability analysis in the healthy subjects while supine (REST) and during 70° head-up tilt test (TILT) in normal condition (B) and while listening activating (Track1) and relaxing (Track2) algorithmic music. RR, RR interval; μ_RR_, RR mean (**a**); σ^2^_RR_, RR variance (**b**); LF, low frequency; LF_a,RR_, absolute power of RR in the LF band (**c**); LF_nu,RR_, normalized power of RR in the LF band (**d**); HF, high frequency; HF_a,RR_, absolute power of RR in the HF band (**e**); HF_nu,RR_, normalized power of RR in the HF band (**f**). Data are expressed as mean ± standard deviation. § indicates *p* < 0.05 REST vs. TILT. * Indicates B vs. Track1 and vs. Track2.

The results of the RR variability analysis for each experimental session (namely B, Track1 and Track2, in REST and TILT) are summarized in Figure 3.

In B, all subjects were characterized by normal μ_RR_ (corresponding to a HR of 71 ± 8 bpm at REST and 85 ± 10 bpm during TILT), as well as normal indices of cardiac sympathovagal balance, in both REST and TILT. Physiologically, from REST to TILT, μ_RR_ decreased, the indices of vagal modulation directed to the sinus node, i.e., HF_a,RR_ and HF_nu,RR_, also decreased, while the indices of cardiac sympathetic activity, i.e., LF_a,RR_ and LF_nu,RR_, increased. Accordingly, the LF/HF was 2.90 ± 1.15 and 5.39 ± 5.82 in REST and TILT, respectively.

**Figure 4:**
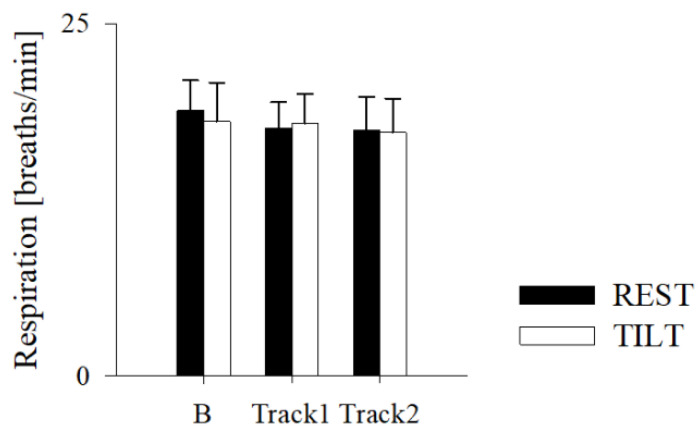
Results of the respiratory rate estimation in the healthy subjects while supine (REST) and during 70° head-up tilt test (TILT) in normal condition (B) and while listening to Track1 (activating algorithmic music) and Track2 (relaxing algorithmic music). Data are expressed as mean ± standard deviation.

Compared to B, both Track1 and Track2 determined an increase of μ_RR_ at REST, (corresponding to a HR of 71 ± 8 bpm at REST and 67 ± 8 bpm during Track1 and 69 ± 9 bpm during Track2), without modifying σ^2^_RR_, the sympathetic or vagal indices, including the LF/HF (2.47 ± 2.07 for Track1 and 5.42 ± 6.75 for Track2). Of notice, also the respiratory rate remained unchanged (Figure 4).

Similar to B, TILT during Track1 and Track2 induced a decrease of μ_RR_, of HF_a,RR_ and HF_nu,RR_, and an increase of LF_nu,RR_ and LF/HF (8.27 ± 3.44 for Track1 and 13.46 ± 9.88 for Track2). There were no differences between each TILT phase in the three experimental conditions.

Table 3 shows the results of the AP variability analysis. All subjects showed μ_SAP_ and μ_DAP_ within normal range in B, both at REST and TILT.

LF_SAP_, an index of the sympathetic modulation directed to the vessels, increased during TILT, while σ^2^_SAP_ remained unchanged, as it occurs in normal subjects. Compared to B, both Track1 and Track2 had no effects on μ_SAP_, μ_DAP_, σ^2^_SAP_, and LF_SAP_. Similarly, TILT induced an increase of LF_SAP_, during both Track1 and Track2, while μ_SAP_, μ_DAP_, and σ^2^_SAP_ remained unchanged.

Finally, the analysis of the three different recovery periods did not show any significant result.

**Figure 5:**
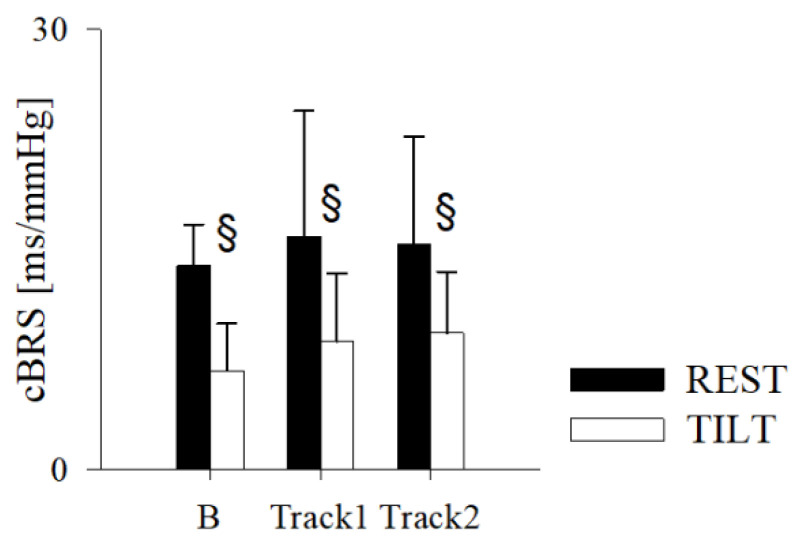
Results of the cardiac baroreflex sensitivity (cBRS) estimation in the healthy subjects while supine (REST) and during 70° head-up tilt test (TILT) in normal condition (B) and while listening to activating (Track1) and relaxing (Track2) algorithmic music. Data are expressed as mean ± standard deviation. § indicates *p* < 0.05 REST vs. TILT.

Figure 5 shows the results of the cBRS. Consistent with the above results, the cBRS was within normal values in B and was not affected by either Track1 or Track2. It decreased from REST to TILT in B, as well as in Track1 and Track2 sessions.

## 4. Discussion

In the present study, a group of healthy subjects underwent an experimental protocol to evaluate their CV neural profile at baseline and in response to algorithmic music listening, consisting of an activating and a relaxing song in a randomized order.

The striking finding is that, compared to baseline, listening to both algorithmic music tracks, composed with activating or relaxing purposes, induced a significant decrease of HR at REST, without affecting AP, breathing rate, and the indices of CV neural modulation. In several studies, it has been reported that music listening leading to emotional arousal is associated with higher HR than listening to tranquilizing music [25,26,27]. In this study, we observed that HR was similar while listening to Track1 and Track2. Indeed, the type of musical stimulus per se utilized in this study could justify such desirable results. The algorithmic music mainly focuses on musical structures and parameters (in relation to therapeutic aims) [12] rather than aesthetic aspects or the emotional engagement, which is considered one of the most important mechanisms involved in the modulation of the ANS regulating HR [3].

Our results also showed that passive orthostatic challenge had a dominant effect on both musical stimuli, as during TILT no differences between Track1 and Track2 were found. In other words, the orthostatic stimulus elicited a physiological response regardless of the music listening.

Indeed, the physiological response to TILT consisted of an increase in HR, together with the expected rise of sympathetic modulation directed to the heart and vessels, and withdrawn of vagal modulation directed to the sinus node [14,17,21,22]. The three recovery periods were characterized by similar HR, AP, and CV autonomic indices.

Therefore, in these healthy subjects featuring a normal CV neural profile at baseline, musical stimulation, either Track1 or Track2, reduced HR, a potentially favorable effect, independently from breathing rate and sympathovagal balance.

In fact, it is well known that a physiological lower HR in healthy populations is associated with lower CV and all-cause mortality [28,29,30]. Similarly, among patients with chronic coronary syndromes, heart failure or hypertension, lower HR is associated with lower overall and CV mortality, and lower CV hospitalizations [31,32,33]. The conceivable underlying mechanisms are multiple, from optimization of metabolic and myocardial oxygen demand to lengthening of the coronary perfusion time and reduced cyclical stretch of the large elastic arteries [34].

Pharmacological lowering of HR has long been recommended and employed with a cardioprotective aim in several conditions [35,36]. However, drug induced bradycardia often implies additional effects, including those on the CV neural regulation, that might be disadvantageous in some conditions. As a relevant example, in hypertension, the use of beta-blockers should be targeted based on the individual’s characteristics and the specific beta-blocker’s properties [37], although measuring HR and managing tachycardia represents a major goal [35]. Again, ivabradine, which inhibits the funny current (*I_f_*) in sinoatrial nodal tissue without affecting cardiac inotropy or systemic vascular resistance can be recommended in chronic coronary syndromes and heart failure [38]. In this perspective, music listening might represent a non-pharmacological intervention aimed at improving outcomes in both healthy and diseased persons.

Furthermore, additional different effects of music listening are conceivable in the case of patients with abnormal CV nervous control. In fact, it is known that in pathological conditions, some interventions may restore some sympathovagal control [39,40], that might be due to the activation of afferent pathway projecting to the medulla oblongata influencing both the sympathetic and parasympathetic branches with distinguishable features when activating the different groups of fibers [41].

Interestingly, in the present study, algorithmic music had no effects on the respiratory rate, thus the HR reduction was independent, unlike what has been demonstrated with the rhythmic activity of music or other practices [42,43]. From this point of view, the use of algorithmic music would allow a proper adjustment and tailoring of the sound stimuli based on the underlying conditions on the one hand and therapeutic goals on the other [12].

It remains to be established which mechanisms may intervene in reducing HR during listening to algorithmic music. It has been previously reported a decrease of HR in young healthy subjects during physiological conditions without changes in cardiac ANS [44]. An endocrine response could be involved, as it is known that music may lower serum cortisol levels with inhibition of CV stress reaction even in the short-term [45,46].

Regardless of the underlying factors, mean HR, and even more nocturnal HR, is associated with increased mortality in several cardiac conditions, and algorithmic music may exert favorable preventive and therapeutic effects.

### Limitations

This study analyzed the acute effect of music listening for a short lapse of time. Chronic and/or repeated exposure to music listening might produce more noticeable effects, perhaps highlighting the differences between activating and relaxing stimuli.

The sample size was limited; therefore, it prevents us from having strong results. For the same reason, we did not analyze the data along the lines of gender and sex. However, as these variables are known to influence the CV responses to external stimuli [42,47], future studies are advocated. In addition to gender, several parameters such as age of the participants or the duration of the baseline period could potentially affect the physiological responses [48,49].

## 5. Conclusions

It is widely accepted that music has therapeutic effects in many neurodegenerative and psychiatric diseases and stress-related disorders [5,8]. Effects on the cardiovascular system [2,3,4,5,6,11,15] have also been described; however, the results of studies on this topic are often inconsistent. Therefore, there is pressing need for systematic high-quality research on the effects of music on the heart in both healthy individuals and patients.

In this study, on a small group of healthy subjects, both the relaxing and activating algorithmic music decreased the HR, without supposedly changing the cardiac sympathovagal balance, arterial blood pressure, vascular sympathetic modulation, baroreflex sensitivity, and the response to the orthostatic challenge, that were all physiological at baseline in this study population. Importantly, no kind of music, neither the activating nor the relaxing one, produced negative effects. Still, some music-induced effects on an already normal ANS might be difficult to detect. Otherwise, different results could be found in patients, in which music could determine a rebalancing of an altered cardiovascular neural profile, for example in the elderly, in neurodegenerative and CV diseases [19,48]. In other words, while music would slightly interfere with a healthy subject’s heart, still lowering the HR in a beneficial way, in patients with a reduced variability of the overall HR or in healthy subjects under particular conditions, such as stress [19] or aging [48], the music could play a more incisive and favorable role.

In this perspective, this study represents an absolute novelty introducing an innovative and standardized music approach based on tailored musical patterns with therapeutic goals. This approach creates considerable potential in the use of musical listening aimed at the care and health of individuals and opens the door to further studies on different categories of patients.

## Figures and Tables

**Table 1:** RR and SAP variability parameters.

μ_RR_ [ms]	Mean of the RR intervals [21]
σ^2^_RR_ [ms^2^]	Variance of the RR intervals [21]
LF_a,RR_ [ms^2^]	Absolute power of RR series in the low frequency band (LF, 0.04–0.15 Hz), index of cardiac sympathetic modulation [17,22]
LF_nu,RR_ [nu]	Normalized power of RR series in the LF band, index of cardiac sympathetic modulation [17,22]
HF_a,RR_ [ms^2^]	Absolute power of RR in the high frequency band (HF, 0.15–0.4 Hz), index of cardiac vagal modulation [14,21]
HF_nu,RR_ [nu]	Absolute power of RR in the high frequency band, index of cardiac vagal modulation [14,21]
LF/HF	Index of cardiac sympathovagal balance [21]
μ_SAP_ [mmHg]	Mean of the SAP values [21,22]
μ_DAP_ [mmHg]	Mean of the DAP values [21,22]
σ^2^_SAP_ [mmHg^2^]	Variance of the SAP [21,22]
LF_SAP_ [mmHg^2^]	Absolute power of SAP series in the low frequency band (LF, 0.04–0.15 Hz), index of vascular sympathetic modulation [21,22]
cBRS [ms/mmHg]	Cardiac baroreflex sensitivity [23,24]

**Table 2:** Demographic and clinical features of the enrolled population.

Age, years	39.2 ± 6.4
Gender, males/females	5/5
BMI, kg/m^2^	22.6 ± 1.4
BMI males, kg/m^2^	23.4 ± 1.3
BMI females, kg/m^2^	21.8 ± 1.1
Sleep per night, hours	6.1 ± 1.0
Occasional smoking, *n* (%)	2 (20)
Regular physical exercise, *n* (%)	8 (80)
Physical exercise, hours/week	4.4 ± 2.4
Regular social activities, *n* (%)	8 (80)
Social activities, hours/week	7.1 ± 4.4

Data are presented as mean ± standard deviation or number (percentage).

**Table 3:** Results of the arterial pressure variability analysis.

	B		Track1		Track2	
	REST	TILT	REST	TILT	REST	TILT
μ_SAP_, mmHg	119 ± 8	116 ± 10	112 ± 16	115 ± 9	113 ± 8	110 ± 15
μ_DAP_, mmHg	76 ± 11	77 ± 12	72 ± 14	77 ± 12	74 ± 8	76 ± 7
σ^2^_SAP_, mmHg^2^	23.7 ± 15.9	38.6 ± 26.7	36.9 ± 33.6	31.1 ± 18.9	22.9 ± 13.5	32.1 ± 20.3
LF_SAP_, mmHg^2^	0.91 ± 1.05	2.88 ± 3.09 ^§^	0.66 ± 0.33	3.42 ± 4.19 ^§^	0.75 ± 0.52	2.82 ± 2.75 ^§^

Results of the arterial pressure variability analysis in the healthy subjects while supine (REST) and during 70° head-up tilt test (TILT) in normal condition (B) and while listening to activating (Track1) and relaxing (Track2) algorithmic music. SAP, systolic arterial pressure; DAP, diastolic arterial pressure; μ_SAP_, SAP mean; μ_DAP_, DAP mean; σ^2^_SAP_, SAP variance; LF, low frequency; LF_SAP_, absolute power of SAP in the LF band. Data are expressed as mean ± standard deviation. ^§^ indicates *p* < 0.05 REST vs. TILT.

## Data Availability

Data are available upon reasonable request to the corresponding author.

## Supplementary Materials

The following are available online at https://www.mdpi.com/article/10.3390/jpm11111084/s1, Audio S1: algorithmic music Track1, Audio S2: algorithmic music Track2.
